# Patient and provider perspectives inform an intervention to improve linkage to care for HIV patients in Ukraine

**DOI:** 10.1186/s12913-018-2885-4

**Published:** 2018-01-30

**Authors:** Tetiana Kiriazova, Oleksandr Postnov, Trista Bingham, Janet Myers, Timothy Flanigan, Charles Vitek, Oleksandr Neduzhko

**Affiliations:** 1 Ukrainian Institute on Public Health Policy, 4, Malopidvalna Street, Of.6, Kyiv, 01001 Ukraine; 2 Division of Global HIV and Tuberculosis, Centers for Global Health, U.S. Centers for Disease Control and Prevention, 1600 Clifton Rd, Atlanta, GA 30333 USA; 3 Division of Prevention Science, University of California, San Francisco, 550 16th Street, 3rd Floor, San Francisco, CA 94158 USA; 4 Warren Alpert Medical School, Brown University, 222 Richmond St, Providence, RI 02903 USA; 5 Division of Global HIV and Tuberculosis, Centers for Global Health, U.S. Centers for Disease Control and Prevention, 4 Igor Sikorskiy Street, Kyiv, 04112 Ukraine

**Keywords:** HIV care, Linkage to care, ARTAS, Intervention, Ukraine

## Abstract

**Background:**

Engagement with HIV medical care is critical to successful HIV treatment and prevention efforts. However, in Ukraine, delays in the timely initiation of HIV treatment hamper viral suppression. By January 01, 2016, only 126,604 (57.5%) of the estimated 220,000 people living with HIV (PLWH) had registered for HIV care, and most (55.1%) of those who registered for HIV care in 2015 did that at a late stage of infection. In the US, Anti-Retroviral Treatment and Access to Services (ARTAS) intervention successfully linked newly diagnosed PLWH to HIV services using strengths-based case management with a linkage coordinator. To tailor the ARTAS intervention for Ukraine, we conducted a qualitative study with patients and providers to understand barriers and facilitators that influence linkage to HIV care.

**Methods:**

During September–October 2014, we conducted 20 in-depth interviews with HIV-positive patients and two focus groups with physicians in infectious disease, sexually transmitted infection (STI), and addiction clinics in Dnipropetrovsk Region of Ukraine. Interviews and focus groups were audio-recorded and transcribed verbatim. We translated illustrative quotes into English. We used thematic analysis for the data analysis.

**Results:**

Participants (20 patients and 14 physicians) identified multiple, mostly individual-level factors influencing HIV care initiation. Key barriers included lack of HIV knowledge, non-acceptance of HIV diagnosis, fear of HIV disclosure, lack of psychological support from health providers, and HIV stigma in community. Responsibility for one’s health, health deterioration, and supportive provider communication were reported as facilitators to linkage to care. Expected benefits from the case management intervention included psychological support, HIV education, and help with navigating the segmented health system.

**Conclusions:**

The findings from the study will be used to optimize the ARTAS for the Ukrainian context. Our findings can also support future linkage-to-care strategies in other countries of Eastern Europe and Central Asia.

## Background

Engagement with HIV medical care is critical to the success of HIV treatment and prevention efforts. In the past decade, the global community has made great strides in scaling up access to HIV care and treatment. Between 2002 and 2012, access to antiretroviral therapy (ART) increased from 300,000 people to 9.7 million in low- and middle-income countries [1]. This rapid scale-up has resulted in a substantial decline in the number of HIV-related deaths and new HIV infections.

While the HIV epidemic is declining in many global settings, Eastern Europe and Central Asia are regions where the rate of new HIV diagnoses continues to increase. In Ukraine, delays in initiation of HIV treatment hamper viral suppression. As of January 01, 2016, only 126,604 of the estimated 220,000 people living with HIV (PLWH) in Ukraine had registered for HIV care at the AIDS Centers [2]. In addition to late diagnosis, delays between diagnosis and entry to HIV care deny individuals the benefits of early treatment. In 2015, 55.1% of newly registered PLWH enrolled in HIV care at the III-IV clinical stages of HIV infection, as defined by the World Health Organization [2, 3].

Delays in linkage to care can result from patient-related factors (fear, substance use, etc.) and system-level factors (poor availability of services, lack of case management, etc.) [4–7]. These barriers lead to suboptimal outcomes for linkage to care and retention in HIV care and treatment – all of which contribute to poor long-term health outcomes and inability to achieve epidemic control in Ukraine.

Several evidence-based interventions were shown to improve linkage to care among recently diagnosed PLWH. In the USA, the Anti-Retroviral Treatment and Access to Services (ARTAS) intervention, sponsored by the Centers for Disease Control and Prevention (CDC), successfully linked newly diagnosed HIV-positive individuals to HIV services using strengths-based case management with a linkage coordinator (LC) [8]. However, it was unclear how well this intervention could be translated to resource-constrained settings such as Ukraine, where HIV care is delivered strictly through specialized AIDS Centers and their affiliates. Thus, we conducted qualitative interviews and focus groups with HIV-positive patients and health care providers (HCP) to learn about factors impacting linkage to and retention in care, so that ARTAS could be adapted for use in the Ukrainian context.

## Methods

### Design

From September to October 2014, we conducted: 1) semi-structured, in-depth interviews with 20 adult participants recently diagnosed with HIV and 2) two focus groups (FG) with HCPs (*n* = 14, seven in each group). As in Ukraine with its concentrated HIV epidemic, most at risk populations often refer for the medical services to the specialized health care facilities (SHCF), which provide care and treatment for infectious disease, sexually transmitted infections (STI), and substance abuse, we recruited HIV-positive participants from these three types of SHCFs located in Dnipropetrovsk Region of Ukraine. For the FG discussions, we recruited HCPs from the same SHCFs and from three AIDS Centers located in the region.

This study obtained ethical approval from the Institutional Review Board of the Ukrainian Institute on Public Health Policy and clearance on human subjects review from the CDC in Atlanta, USA.

### Study setting

Dnipropetrovsk Region, located in central Ukraine, has one of the highest burdens of HIV in the country with an HIV prevalence of 774.0 infected persons per 100,000 (as of January 01, 2016), compared with the national prevalence of 297.2 per 100,000 [2]. Standard of care in the SHCFs typically involves HIV counseling, testing and referral (in case of a positive HIV test result) to an AIDS Center, where enrollment in care takes place and free HIV services are provided.

### Study participants

Patients were eligible for the study if they were fluent in Russian or Ukrainian, were 18 years or older, lived in Dnipropetrovsk Region, tested HIV-positive within past 6 months at one of the participating SHCFs, and gave informed consent to be interviewed. We recruited patients via direct referral from their providers. Participants were compensated 160 Hryvnas (equivalent to $20 US) for their time and transportation expenses.

Eligible HCPs were physicians at SHCF who refer their patients for HIV testing and to the AIDS Center if necessary, or physicians at the AIDS Centers responsible for enrollment in HIV care. HCPs in the focus groups consented to participate and were recruited by referral from chief physicians of participating facilities. HCPs did not receive monetary compensation for being in the study.

### Data collection

#### Semi-structured interviews and demographic questionnaire

We collected demographic data of patient participants with a self-administered questionnaire. The semi-structured interviews explored factors (barriers and facilitators) thought to influence patient HIV service utilization, as well as patients’ perceptions of the ARTAS linkage-to-care intervention described to them; interviews lasted 30–40 min.

#### Focus groups

We conducted focus groups with HCPs to explore their opinions on barriers and facilitators to HIV care for HIV-positive patients of SHCFs. Following a description of the ARTAS intervention, we asked providers’ opinions of how case management may assist linking patients to HIV care, and discussed implementing ARTAS at health care settings in Ukraine. Each FG lasted 1.5–2 h.

Interviews and FGs were conducted in Russian, audio-recorded and transcribed verbatim. We translated illustrative quotes into English.

### Data analysis

We used thematic analysis to analyze the study data [9]. TK and ON reviewed all transcripts, identified emerging themes and domains, and developed a codebook for analysis. Then investigators revised the codes, updated the coding frame as new codes emerged, and organized codes into broader themes [9, 10].

## Results

### Participant characteristics

#### HIV-positive patients

We screened 23 patients to enroll 20 eligible participants - four at Dnipropetrovsk STI Dispensary, nine at Dnipropetrovsk Narcology Dispensary, and seven at Kryvyi Rig Infectious Disease Clinic. Participants’ median age was 38.5 years (interquartile range (IQR) = 32.3–45.3); there were nine men (45.0%). Socio-demographic characteristics of the interview participants are shown in Table 1.

**Table 1:** Socio-demographic characteristics of the HIV-positive semi-structured interview participants (*n* = 20) in Dnipropetrovsk, Ukraine

Characteristics	*N* = 20	#	%
Gender	Male	9	45.0
	Female	11	55.0
Age	38.5 (32.3–45.3)^a^	20	
Marital status^b^	Married	0	0
	Living with partner / In stable relationship	9	45.0
	Widow / Widower / Divorced	10	50.0
	Never married	1	5.0
Education	10 grade or less	2	10.0
	11 grade / Vocational school	13	65.0
	College / Higher education	5	25.0
Employment at the time of the interview	Employed (full-time / part-time)	12	60.0
	Looking for a job	3	15.0
	Homemaker	2	10.0
	Other	3	15.0
Level of poverty (“*In past year, how often you did not have money for basic things such as food or housing?*”)	Daily/ Weekly	2	10.0
	Monthly	2	10.0
	Sometimes	8	40.0
	Never	8	40.0
Having permanent place of residence in past 30 days	Yes	16	80.0
	No	4	20.0

^a^Median (IQR)

^b^More than one variant of the response was possible; however, if a respondent marked both “divorced” and “living with a partner”, we categorized the response as “living with a partner”

#### Health care providers

Twenty HCPs were eligible for the study and provided informed consent; of them, 14 HCPs without a work schedule conflict participated in the FGs. Participants represented Dnipropetrovsk Regional and City AIDS Centers, Dnipropetrovsk STI and Narcology Dispensaries, Kryvyi Rig AIDS Center and Infectious Disease Clinic; they were 12 physicians and 2 department chief physicians. Participants’ median age was 40.5 years (IQR = 36.8–42.8); there were two men (14.3%).

Using data obtained from patients and providers, we summarized their perceptions of barriers and facilitators to HIV care, and opinions and expectations following a description of the ARTAS linkage-to-care intervention.

### Barriers to receiving HIV care

Describing factors that prevent people from linking to HIV care, study participants named multiple, mostly individual-level barriers.

### Lack of knowledge about HIV infection / HIV care

Patients described lack of HIV knowledge, including of ART and its side effects, as one of the main barriers to seeking HIV care. They mostly associated HIV care with taking medications (“pills”). Some respondents talked about HCP unwillingness to provide information about treatment of HIV infection and opportunistic infections.

*“I think HIV care is about pills and some help. Good nutrition, first of all. Abstinence from drugs. These are main things in HIV care. People who have HIV should eat well and take pills. There is some therapy, right? This is what I know”. (STI Dispensary, male patient)*

*“Side effects! I need to know more about them. For me it is important to understand what harm I will get from that therapy. Side effects - there is a whole list of them written, as a newspaper, with fine letters. Well, I was using drugs for many years, my health is damaged, I am afraid to start therapy... And physicians do not explain anything. They put it this way: either you take it* [ART] *and you are fine, or you do not, and you die”. (Narcology Dispensary, male patient)*

Providers emphasized that patients lack information about benefits of HIV services and that HIV information is typically presented “in a negative way.”

*“Lack of information! Both about HIV and about available services and why they are needed. There should be commercials about positive aspects. Because if you have AIDS, then - the line is drawn, and - only black color… There should be some bright colors, so that people see that it is not the end and there is a way out”. (Provider)*

Lack of posttest counseling emerged as one of the reasons behind patients’ poor knowledge about HIV care. However, providers from the same facilities described their communication with patients who tested HIV-positive as “productive.”

*“They invited me in the room, and just – ‘You have HIV, don’t worry’, that’s all. Well, I went speechless…* (Interviewer: Did they explain you anything about HIV infection?) *No, no, they only asked me to sign a document that I would not have sex with anyone.* (Interviewer: Did you sign such document?) *Yes, I did. Well, I realize how I got infected, but besides that I do not know anything.*” *(Infectious Disease Clinic, female patient)*

*“I conduct a counseling with patients, who learned about their HIV infection for the first time. In our clinic this kind of work is done on a very good level”. (Provider)*

### Patients’ initial rejection of HIV diagnosis

Another common theme reported by both patients and providers was patients’ non-acceptance of HIV status, resulting in delays of HIV care seeking and HIV status disclosure.

*“If I were not hospitalized, I would never learn my HIV diagnosis… Honestly, I am still in a shock, because even now I do not believe for hundred percent. It’s like a nightmare - I never thought this might happen to me…” (Infectious Disease Clinic, male patient)*

*“Sometimes a person needs time to accept* [HIV] *diagnosis. Sometimes one month, or two, or three. And of course, such person is not ready to come home and immediately tell them about his diagnosis or to do anything about it”. (Provider)*

### HIV-related stigma

Participants identified HIV-related stigma and negative attitudes toward PLWH as a major barrier that prevented PLWH from seeking medical care. However, while patients talked about stigmatizing attitudes and violations of their rights in health facilities, HCPs tended to talk about HIV stigma “in society” in general.

“*I know a guy, who has HIV. His leg hurt. He came to the hospital and told about his* [HIV] *status. They refused to treat him – ‘Sorry, we won’t take care of you’. This happened. But health professionals should be informed! So what, if he has HIV – let his leg break off? For me, it is about lack of knowledge or unwillingness to understand. I mean primary care clinics, ordinary physicians and nurses.” (Narcology Dispensary, male patient)*

*“If our society treated people with HIV infection like all others, it would help them seek HIV care. It would be easier for them, much easier”. (Provider)*

*“In all instructions that we receive, it is so well written how to communicate with HIV-positive people and how these people should be treated in society… But how are they perceived in society? Extremely, extremely negatively. When a person learns that he has AIDS, he understands that he will be offended and abused”. (Provider)*

### Fear of HIV status disclosure

Almost all patients reported fear of HIV status disclosure; providers confirmed such fear as a barrier to HIV care. They reported that a person can be fired after his HIV diagnosis becomes known to his employer. For this reason, doctors avoid writing an HIV-related code in a person’s sick leave, to protect patients from confidentiality breach and from discrimination at the workplace.

“*Family do not know. No one knows. This is difficult! Sometimes you think of telling someone… But my family will not understand. I am afraid that they will not accept me. They will put me in a hospice. And it will be the end.” (Narcology Dispensary, female patient)*

“*When patients learn of their status, they pleadingly ask, ‘Will this be written in my sick leave?’ You know, unfortunately, our society... At a factory, at a mine, when they learn about someone’s HIV diagnosis, they will find a thousand reasons to fire this person. So we don’t write HIV-related code. We document a sick leave with a concomitant disease diagnosis – pneumonia or herpes… We protect patients”*. *(Provider)*

### Facilitators of HIV care entry

Patients and providers recognized several potential facilitators for successful linkage to HIV care, such as having such poor health that treatment was an urgent need; patient communication skills; and supportive attitude from health providers.

### Experiencing poor health

While asymptomatic patients often reported they “*currently do not need*” HIV care, poor health and HIV infection symptoms were frequently mentioned as motivators for seeking care.

(Interviewer: What could facilitate your linkage to HIV care?) “*Probably bad health condition. When I would not be able to walk. Completely. To walk, to breathe… When I could just drop in the street. But as long as I can move, can work, I do not want to go there* [to the AIDS Center].” *(Narcology Dispensary, female patient)*

### Patient-provider communication

For some participants who visited AIDS Center and did not report any difficulties with enrollment, support of HCP was a motivating factor that shaped their care-seeking behavior.

“*In my case, it all happened fast - I learned about my status, they immediately did a test for CD4 cells, for viral load... It began turning like a wheel, I even did not understand. I am already taking therapy, you know! My doctor-infectionist supported me. She told me where to go, what to do, whom to call. I just was doing everything she told me to do. And I did not face any rejections.” (Narcology Dispensary, male patient)*

Similarly, providers mentioned efficient patient-provider communication as a factor that could improve HIV care uptake, especially when a physician tells patients about HIV treatment benefits and availability. One HCP explained, *“A physician should motivate patients and explain why they need medical observation – to have examinations and start HIV treatment in time”*.

### Opioid substitution therapy as a facilitator of HIV care

Providers of narcology care often perceived patients’ “main diagnosis” (addiction-related mental and behavioral disorders) as a barrier to their linkage to HIV care, and drug dependent persons themselves as “*undisciplined*” and different from “*socially normal*” patients. At the same time, providers talked about opioid substitution therapy (OST) as a facilitating factor for drug-using PLWH. Because OST patients visit Narcology clinic daily, they can undergo all examinations onsite, while physicians have time to motivate patients to link to HIV care.

*“OST patients are more connected and more disciplined. If one’s rapid test result is positive, we immediately take blood for ELISA and inform him when the result is confirmed. We can also do CD4 testing... And we have time to motivate the patient to go and register for HIV care.” (Provider)*

### LC as a source of HIV information and psychological support

After the elements of the ARTAS intervention were described to the respondents [11], all agreed that the intervention should focus on linkage coordination at the SHCFs.. Patients expected LC would provide them with psychological support and HIV information, help with navigating the health system and with coordinating clinic visits. They described the benefits of having a person with whom they could discuss their health concerns. Patients saw an accessible health specialist as a source of HIV information that they did not get from a physician. Some mentioned assistance with HIV disclosure as an aspect of such support.

*“Why people do not register* [at AIDS Center]… *Some people just do not know how serious it is. I mean, you need to know that if you make an effort to start treatment, it is much better than to apply at the last minute. Many do not understand this.* [Interviewer: So if someone could explain…] *It would be great.” (STI Dispensary, female patient)*

*“The advantage of case management is that in a face-to-face conversation, a person may ask questions about own health problems, and the educated coordinator will explain everything in a lay language.” (Narcology Dispensary, male patient)*

### Coordination / navigation

Discussing their needs regarding linkage to care, patients liked an opportunity to get assistance, especially during their initial visit to the AIDS Center. Health providers also were supportive about case management intervention; they believed that HIV-positive patients “*should be taken by the hand and directly brought to the AIDS Center*.”

*“Such person is really needed. Because everything is new. If you come to the AIDS Center, where there is a person who knows where to go, what to do with all these papers, it will be much easier for you. Like they told me – you have positive test. What should I do? I do not know… So I go home and live as I lived before. But if you are told - ‘Now you should do this and that’, - it will be different”*. *(Narcology Dispensary, female patient)*

*“In this program the person is not left alone with the problem. The burden of the transportation to the AIDS Center is not put on the patient only. He gets help, he feels support. He knows that someone is thinking of him.* [Altogether] - *Yes! Exactly*!” *(Provider)*

### Health professional as an ideal LC

Among traits of “an ideal linkage coordinator”, patients mentioned reliability, empathy, and a positive attitude toward PLWH. Most participants preferred the LC to be a health professional able to provide reliable HIV information. All agreed with the proposed modification to the ARTAS introducing nurses as LCs, and were unanimous in opinion that a trained nurse could be an ideal LC understanding where to refer patients and what information to provide.

*“Such coordinator needs a correct approach to build contact with a patient. To give convincing arguments on benefits from HIV treatment… To be knowledgeable, and to be a good psychologist, because people are different.” (Narcology Dispensary, male patient)*

*“If a linkage coordinator is a health specialist, this will ensure more respect from clients. They will know they can get reliable information from her.” (Provider)*

### Considerations for successful ARTAS implementation

Most participants did not see any major flaws in the described intervention; their main concern was the inability of an LC to perform her duties if she is overloaded with many patients or her salary is small. Both patients and providers suggested to limit the load of the LC and to motivate her with decent wages.

*“I can see only positive aspects of the proposed model, especially regarding primary care facilities without direct linkages with AIDS Center. There may be some minor flaws, but nothing serious.” (Provider)*

*“A nurse should be trained. It should be a staff member with specific functional duties, getting decent salary.” (Provider)*

*“She may have, let’s say, 10 clients at a time... Once one client connects with HIV care, she takes a new client. It should be a limited load, 10-15 clients at a time, no more.” (Provider)*

“*I think there may be problems, such as a lack of personnel, or small salary, or they will have too many clients to perform their duties in full... Then she* [LC] *may say that she has many patients today and cannot meet with me, and will be available in a week or two… That is what I am afraid of.” (Infectious Disease Clinic, female patient)*

## Discussion

With this qualitative study, we explored barriers and facilitators to HIV care initiation from both patient and provider perspectives. In the interviews and FGs, we also asked about participant views on the feasibility of the proposed ARTAS intervention and about its potential role in linking PLWH to medical care within the Ukrainian context. Respondents described how a LC, a key component of the intervention, could help patients to overcome existing barriers to HIV care entry.

Respondents in our study shared views about multiple, mostly individual-level barriers to HIV care and treatment. These findings support previously reported data about multiple factors affecting HIV care entry [12, 13]. Our findings also echo key barriers reported in previous research, which include postponing HIV care while feeling healthy, poor client–provider communication, and experiences of stigma and discrimination [14–16].

While both patients and providers supported the importance of patient awareness of the main aspects of life with HIV infection, there was a “disconnect” between their opinion on the quality of HIV testing and counseling services provided in the SCHFs. The majority of patients said they had not received HIV posttest counseling, while physicians from the same clinics reported providing quality counseling to all patients with positive HIV test result. Overall, most respondents described feeling dissatisfied with their posttest counseling. These data support conclusions by Garland et al. [17], that training of medical staff to provide more thorough posttest counseling in all HIV test settings may be needed to improve access to HIV care.

Initial rejection or non-acceptance of HIV-positive status emerged as an additional barrier to seeking HIV care, mentioned by both patients and HCPs. These data add to previous findings, which reported denial by individuals of their newly diagnosed HIV status and needing more time to accept HIV diagnosis as the common barrier to HIV care entry [17, 18]. The ARTAS II study in the USA showed that clients who were ready to cope with their HIV-positive status were more likely to enter HIV care [19]. For this reason, quality posttest counseling could minimize the tendency to deny one’s HIV status, providing the individuals who test positive with appropriate information on managing their HIV infection so they will be more likely to take necessary actions. A recent systematic review cited the need for stronger posttest counseling, as well as implementing treatment literacy programs, as necessary steps to improve linkage to care [12].

Both groups of respondents talked about stigmatizing attitudes toward PLHW, especially toward substance-using individuals. However, patients and HCPs perceived stigma in different way: patients talked more about stigmatizing attitudes and violations of their rights at health facilities, while providers described HIV stigma “in the society” in general. Similarly, many HCPs labelled their substanceusing patients as “anti-social people”, different from “normal” (socially well-established) patients; however, providers did not perceive themselves and their attitudes as a source of stigma toward substance-using PLWH. Providers’ opinions of substance-using individuals as less likely to go for HIV care is supported by research [20–22]; a sub-analysis of the ARTAS study [8] showed as well that drug-using clients were not linked to care as effectively as clients with no history of drug use. Therefore, it is important to explore whether intensive case management intervention could promote better linkage to HIV care for patients with drug or alcohol problems within Ukrainian context.

Reportedly, HCPs often underestimate the impact of the underlying emotional barriers that might prevent people from seeking HIV treatment [23]. We found that fear of ART side effects, fear of HIV disclosure, and stigma were among the most common barriers that impeded individuals’ access to HIV services. Stigma and fear of HIV disclosure have been commonly reported psychosocial barriers to HIV care [12]. While societal or community-level barriers, such as stigma in the community, may be more difficult to address [15], patients should expect to find a friendly atmosphere and supportive attitude from HIV providers at public health care facilities. In addition, patient-provider communication and the strengths of provider recommendations of ART have been shown as key factors in patient decision to link to HIV care [24]. However, our findings show that in Ukraine, HIV-positive patients often receive incomplete information, sub-optimal posttest counseling, and limited or non-existent psychological support.

In our study, patients mentioned poor health condition and supportive patient-provider communication as the main potential motivators for their HIV care initiation. A common notion reported previously was that HIV care is for those who are sick [14, 16]; respondents in these studies said they would go into HIV care when they exhibited HIV symptoms. Similarly, most of HIV-positive respondents in our study believed that HIV medical care is necessary only when one is sick. A dissonance between HIV diagnosis and good health condition might lead to rejection of one’s HIV status and thus to avoidance of HIV care. We found that the decision to postpone HIV care until one is sick was reinforced by a lack of knowledge about ART and its side effects, as well as by fear of HIV disclosure.

Our qualitative study findings will inform intervention modifications (Table 2) prior to its piloting and to conducting a randomized controlled trial (RCT) in three high HIV prevalence regions of Ukraine. We will make curriculum adjustments and develop additional training components and role-play activities based on our interview and focus group findings, with an increased focus on providing HIV information and empathetic communication to the intervention clients.

**Table 2:** Adjustments to make to the ARTAS intervention based on qualitative interviews with PLWH and focus groups with HCPs, Dnipropetrovsk, Ukraine

Results of the interviews	Adjustments to the ARTAS intervention
Low education level of patients	LCs will be trained to provide HIV information in lay language.
Sub-optimal posttest counseling; incomplete HIV information	More information on life with HIV infection will be provided to patients.
Beliefs that only symptomatic PLWH need medical care	LCs will discuss with patients the importance of HIV care at every stage of disease, addressing their reluctance to engage in HIV care while feeling healthy.
Lack of psychological support	LCs will deliver empathy and psychological support as the part of the intervention.
Fear of HIV status disclosure to partners and family members	LCs will be trained to role-play with intervention participants practicing their HIV disclosure skills.

In general, all study participants supported the introduction of the ARTAS intervention to improve linkage to HIV medical care. Interviewees expected the LC to provide psychological and moral support, as well as to provide them with HIV information and answer their health-related questions. Therefore, both patients and HCPs preferred LCs to be health workers rather than peer counsellors without medical background.

## Limitations

One limitation of this study is that we recruited our participants from health care facilities (infectious disease, STI and narcology clinics); therefore, people who have not established any links with health care system might perceive different barriers to engagement with medical care. Another limitation is that selecting a limited number of participants from three SHCFs and three AIDS Centers from one particular region might limit generalizability of data to inform the intervention for the whole country. However, epidemiologic trends in the HIV epidemic (which are similar across the country) and the standardization of Ukrainian health care delivery across all regions lets us believe that our results will apply broadly. In addition, we will solicit feedback from larger groups of stakeholders before studying the effectiveness of the tailored ARTAS in the next study phase, using an RCT, in three regions of Ukraine.

## Conclusions

Both patients and providers welcomed the introduction of the ARTAS intervention as a tool to help patients overcome existing barriers to HIV care. The results of our study suggest that the ARTAS case management intervention for engaging people in HIV medical care should be tailored to Ukrainian context to improve its effectiveness. We will use the findings from our qualitative study to optimize the planned linkage-to-care intervention prior to its piloting and to randomized clinical trial implementation. In addition, the findings can be used to support additional strategies (such as improving quality of HIV posttest counseling and stigma reduction campaigns) that may further improve the linkage-to-care context in Ukraine and in other countries of Eastern Europe and Central Asia.

## Abbreviations

AIDS: Acquired immune deficiency syndrome
ART: Antiretroviral therapy
ARTAS: Anti-retroviral treatment and access to services
CDC: Centers for disease control and prevention
FG: Focus group
HCP: Health care provider
HIV: Human immunodeficiency virus
IQR: Interquartile range
LC: Linkage coordinator
OST: Opioid substitution therapy
PLWH: People living with HIV
SHCF: Specialized health care facility
STI: Sexually transmitted infection
